# Functional neuroimaging: the adaptive mechanisms in migraine

**DOI:** 10.1186/1129-2377-16-S1-A6

**Published:** 2015-09-28

**Authors:** Gioacchino Tedeschi, Antonio Russo, Alessandro Tessitore

**Affiliations:** Department of Medical, Surgical, Neurological, Metabolic and Aging Sciences, Second University of Naples, Naples, Italy; MRI Research Center SUN-FISM, Second University of Naples, Naples, Italy; Institute for Diagnosis and Care “Hermitage Capodimonte”, Naples, Italy

Migraine is a common neurological disorder characterized by a primary brain dysfunction subtended by an episodic activation and sensitization of the trigemino-vascular pain pathway. Although migraine attacks are clinically well-defined, the underlying pathophysiology of the more complex migraine scenario is largely unknown. Functional brain changes, that occur because of repeated migraine attacks, may be explained through a maladaptive feedforward allostatic cascade model. The fundamental assumption is that “the brain is a central organ of stress”[1], able to detect stressful or potentially stressful situations and react in form of behavioural and/or physiologic responses. These brain responses, mediated by the autonomic nervous system and neuroendocrine mechanisms, could be either adaptive or maladaptive. In this context, allostasis is the ability to protect the body through adaptation[2]. Contrariwise, the allostatic load and overload, resulting from repeated stress and/or allostasis[3], refers to the wear and tear on the systems that normally support adaptation. Compelling evidence suggests that the brain of migraine patients is significantly different from healthy controls. Some of these differences are related to abnormal cortical activity during painful stimulations, suggesting an “adaptive” response observed in the course of moderate or high noxious trigeminal stimulations. Conversely, other differences relate to abnormality in responses that should be adaptive but become impaired or maladaptive, such as altered brainstem processing. More recently, a decreased functional connectivity was demonstrated within the fronto-parietal network (FPN) in patients with migraine without and with aura in the absence of clinically relevant executive deficits. FPN represents the neural substrate of executive functions and FPN functional abnormalities might be a part of a complex cascade that terminates in a migraine attack. Specifically, FPN functional connectivity changes could underlie a defective executive functioning in which high-demanding conditions cannot be correctly processed and solved by patients with migraine, escalating the brain adaptive response, likely resulting in a risk factor for headache attacks. In conclusion, we believe that in migraine an internal state of dysregulation creates an allostatic load with maladaptive consequences on brain, behaviour, physiological regulation and systemic physiology that progress in a feedforward cascade. Thus, we suggest that migraine should be considered a brain disease and not simply a recurrent acute pain syndrome.
